# A genetic and clinical risk factor algorithm to aid in identifying new cases of chronic kidney disease from the general population

**DOI:** 10.3389/fgene.2026.1799312

**Published:** 2026-07-09

**Authors:** Graham Rodwell, John P. A. Ioannidis, Stuart K. Kim

**Affiliations:** 1 Division of Nephrology, Palo Alto Medical Foundation, Palo Alto, CA, United States; 2 Department of Medicine, Stanford Prevention Research Center, Stanford, CA, United States; 3 Department of Biomedical Data Science, Stanford University School of Medicine, Stanford, CA, United States; 4 Department of Statistics, Stanford University School of Humanities and Sciences, Stanford, CA, United States; 5 Department of Developmental Biology, Stanford University Medical Center, Stanford, CA, United States

**Keywords:** chronic kidney disease, genetic testing, kidney disease - diagnosis, personalized and precision medicine, polygenic risk score

## Abstract

**Purpose:**

The purpose of this study is to develop a genetic test to aid in diagnosing chronic kidney disease (CKD). One challenge in treating CKD is that 80%–90% of people with it are undiagnosed and thus do not access healthcare promptly. The problem arises because early-stage CKD has no overt symptoms, and the current policy is to perform diagnostic tests only when accompanied by risk factors such as old age, hypertension, and diabetes.

**Methods:**

This study describes the development of the RICK (RIsk for Chronic Kidney disease) algorithm that employs a polygenic risk score for CKD plus clinical risk factors to identify people at risk.

**Results:**

In data from the United Kingdom biobank, those in the top decile of RICK have a ten-fold increased risk of CKD, and approximately 49% of all those with CKD are included in this decile. Furthermore, targeted creatinine testing for those in the highest RICK decile would potentially increase the number of individuals diagnosed with CKD by 7.4%. However, the RICK algorithm adds little value for detecting CKD defined by elevated uACR (albuminuria).

**Conclusion:**

Using RICK to selectively test those in the general population with highest risk may help in the early identification of CKD and facilitate early access to renal healthcare. The effectiveness and cost-effectiveness of such testing require further study.

## Introduction

Chronic kidney disease (CKD) is defined as a depression in the filtration function of the kidneys and/or the leak of serum protein into the urine (The EMPA-KIDNEY Collaborative Group, 2023). Using the widely accepted definitions of CKD advocated by the Kidney Disease Outcomes Quality Initiative and the Kidney Disease: Improving Global Outcomes CKD Work Group, the prevalence of CKD is estimated to be approximately 11% of the population (Glassock et al., 2017; Kovesdy et al., 2023). This prevalence is increasing and is closely associated with age, diabetes, and hypertension (Chen et al., 2019). Once established, CKD tends to progress regardless of cause and leads to an extensive and growing disease burden, both with associated cardiovascular illness and with the eventual outcome of end-stage renal disease, managed either with dialysis or organ transplant.

Patients with early-stage CKD are typically diagnosed either by estimating their glomerular filtration rate (eGFR) or their urinary albumin to creatinine ratio (uACR), where kidney disease is defined when eGFR is below 60 mL/min/1.73 m^2^ or uACR >30 mg/g. However, people with early-stage CKD typically do not exhibit overt symptoms, and screening is usually performed only on patients with risk factors such as advanced age, hypertension, or diabetes (Chen et al., 2019). The majority of people with early-stage CKD go undiagnosed (Dharmarajan et al., 2017; Shang et al., 2021). With data supporting a benefit from the modification of risk factors and with a growing armament of therapeutics that may slow disease progression, the early identification of CKD could have a significant impact in the management of both individual patients and healthcare populations (Luyckx et al., 2020).

The heritability of CKD has been estimated from family studies to be between 30% and 75% (Akrawi et al., 2018; O’Seaghdha and Fox, 2011; Satko and Freedman, 2005; Wu et al., 2017). Monogenic kidney diseases (such as polycystic kidney disease caused by mutations in the *PKD1* and *PKD2* genes) are known causes of CKD (Dahl et al., 2023; Groopman et al., 2019; Schott et al., 2024; Connaughton et al., 2019; Snoek et al., 2022). Monogenic kidney diseases are relatively rare and rely on mutations with large effects in a single gene. In contrast, genetic risk for CKD can also be estimated using polygenic risk scores (PRSs), which are algorithms that contain numerous DNA markers. Each DNA site has a miniscule contribution to CKD risk, but all DNA sites collectively hold large amounts of information. Several PRSs have been developed for eGFR or uACR (Khan et al., 2022; Sinnott-Armstrong et al., 2021; Tanigawa et al., 2022; Yu et al., 2021).

Genetic testing could benefit renal health in at least two ways. First, it could help identify new cases of CKD from those who are undiagnosed, facilitating selective screening and enabling early access to renal healthcare. Second, it could provide information that may impact the management of patients already known to have CKD. Previous research has suggested that the genetic testing of patients with mild CKD may aid in identifying those who are most likely to progress to kidney failure (Steinbrenner et al., 2023), (Bakshi et al., 2023).

In this study, we explore how genetic screening could identify those with undiagnosed or undocumented cases of CKD who may benefit from early intervention. Next, we evaluate whether a genetic test for CKD provides information about its severity (stage) for those who have already been diagnosed.

## Materials and methods

### United Kingdom Biobank cohort

Genotype data were obtained from the v3 release of the United Kingdom Biobank (Bycroft et al., 2018). United Kingdom Biobank electronic healthcare records are available for 469,203 individuals and include data until June 2019. Genotype data are imputed centrally by the United Kingdom Biobank with IMPUTE2 using the Haplotype Reference Consortium and the United Kingdom10k+ 1000GP3 reference panels (Howie et al., 2011). Imputed SNPs were excluded if they had an IMPUTE2 info score <0.4. Individuals were excluded if they were outliers based on genotyping missingness rate or heterogeneity, whose sex inferred from the genotypes did not match their self-reported sex, who withdrew from participation, or who were not of European ancestry. The purpose of restricting individuals to those with European ancestry is to reduce population stratification in the study. Genetic variants were excluded that failed quality control procedures in any of the genotyping batches, that showed a departure from a Hardy–Weinberg of P < 10^−50^, or that had a minor allele frequency < 0.001.

### Cohorts with renal function data

The demographics and clinical characteristics of the 72,562 participants who had both eGFR and uACR measurements are compared to those of the 243,703 who had eGFR measurements in Supplementary Table S4. The 72,562 had lower rates of female sex and higher rates of smoking than the 243,703 cohort, but the differences were relatively modest in absolute magnitude. Sex, age, body mass index (BMI), systolic blood pressure (SBP), smoking status, low-density lipoprotein (LDL), and glycated hemoglobin (HbA1C) were recorded by the United Kingdom Biobank during the initial assessment visit (2006–2010) at which participants were recruited and consent given.

### CKD definitions

Diagnosed cases of CKD (CKD-Diagnosed) were identified based on clinical diagnoses captured in the electronic health records by the United Kingdom Biobank. The *International Classification of Disease*, Tenth Revision (ICD-10), Read v2, or Read v3 codes were used to identify cases of CKD (Supplementary Table S3). The electronic medical records originate from a diverse array of healthcare systems. Data from some healthcare systems may be incompletely represented in the United Kingdom Biobank, so some participants may not have a diagnosis because their EHR data was not included in it. The date by which CKD was diagnosed was not available from the electronic medical records. Since CKD is associated with hypertension and hypercholesterolemia, some patients could receive treatment for hypertension and hypercholesterolemia rather than CKD explicitly. These patients were not included in CKD-Diagnosed because they are not explicitly treated for having CKD. Other patients may be included in the GP clinical database but have READ codes that are missing because they transitioned from one outpatient center included in the United Kingdom Biobank to another that was not; these patients would be inappropriately excluded from CKD-Diagnosed due to incomplete GP clinical records. However, the exact number of such transitions has not been quantified but is estimated to be low. CKD cases based on eGFR (CKD-G) were defined as eGFR <60 mL/min/1.73 m^2^. eGFR was calculated using the CKD-Epi 202 equation from age, sex, race, and serum creatinine measurements extracted from the United Kingdom Biobank using measurements that were ascertained during the initial assessment visit (2006–2010) (Levey et al., 2006). CKD cases based on either eGFR or uACR (CKD-GA) were defined as eGFR <60 mL/min/1.73 m^2^ or uACR >30 mg/g. The codes used to define mild CKD and severe CKD are shown in Supplementary Table S3.

### Calculating GFR PRSs from the United Kingdom Biobank

PRSs for GFR and uACR were found by searching the PGS catalog for “GFR” or “uACR” in January 2024 (Lambert et al., 2021). The PRSs from Yu et al. (2021), Khan et al. (2022), Sinnott-Armstrong et al. (2021), and Tanigawa et al. (2022) used cohorts that overlapped with United Kingdom Biobank 35%, 0%, 100% and 100%, respectively (Chang et al., 2015). Scores were generated for each participant using Plink2 with the coefficients from the original publication (Chang et al., 2015). For SNPs with missing data, an average allelic dosage was calculated based on the minor allele frequency for that SNP.

### Development of the RICK algorithm

The RICK algorithm was generated from a total of 243,703 participants of European ancestry with complete data (seven clinical risk factors (CRFs) and GFR PRSs) from the United Kingdom Biobank (Supplementary Table S4). The RICK algorithm was developed in R using a general linear model to fit either eGFR, CKD-G, or CKD-Diagnosed as a response with sex, age, BMI, blood pressure, smoking status, LDL, HbA1C, and GFR PRS as predictors. In addition to fitting models using blood pressure and HbA1C as predictors with continuous values, alternative models were fitted using hypertension and diabetes as discrete predictors; as expected, stepwise regression showed that the alternative models performed poorly compared with models using predictors with continuous values. In addition to the seven CRFs, we evaluated levels of HDL, albumin and cystatin C, and diabetes diagnosis as potential predictors as these risk factors are also known to be associated with kidney disease. We found that the HbA1C level was more informative than a diagnosis of diabetes. Stepwise regression showed that HDL and albumin levels were not statistically significant in predicting eGFR. Patients with a cystatin C measurement typically also had a serum creatinine measurement, in which case using the RICK algorithm to estimate eGFR based on serum creatinine would not be necessary. Hence, diabetes diagnosis and levels of HDL, albumin, and cystatin C were not included in the RICK algorithm.

Some of the clinical risk factors used in the RISK algorithm may be correlated—an issue known as multicollinearity. This does not necessarily affect the model’s ability to predict outcomes; however, its interpretive value (understanding the contribution of individual predictors) is compromised.

The model used ten-fold cross validation, which means that it was trained on 90% of the cohort and then tested on the remaining 10%. This process was repeated ten times, and then the results were averaged together. The model contains coefficients for intercept, each of the CRFs, and the PRSs (Supplementary Table S2). The “cv.glm” function in R’s boot package provided cross-validation results via the delta output. The delta showed that the adjusted prediction error (224.0715) was very close to the raw prediction error (224.0655), which suggests a reliable model.

Values for height, weight, and serum creatinine were determined by the United Kingdom Biobank and were used to calculate BMI and eGFR (with the CKD-Epi 202 equation). Adding uACR as a risk factor to the RICK algorithm made little or no improvement to its correlation with eGFR or the AUC for CKD-Diagnosed. Specifically, uACR was added to the RICK model and was compared with RICK using 72,881 participants (i.e., those with both eGFR and uACR data). The model with uACR had a correlation to eGFR of 64% and an AUC with CKD-Diagnosed of 0.799, compared to a correlation of 0.63 and AUC of 0.797 for RICK by itself.

The performance of each PRS in predicting eGFR and CKD in the United Kingdom Biobank cohort was assessed using two independent measures: 1) correlation coefficient and variance explained and 2) area-under-the-curve (AUC). Correlation measures whether the algorithm and eGFR trend in the same direction, where 100% indicates perfect agreement and 0% indicates no relationship. Variance measures how well the algorithm predicts eGFR and is derived by squaring the correlation, where 100% indicates that eGFR can be precisely calculated and 0% indicates that there is no information about eGFR. AUC assesses how well the algorithm classifies participants as either CKD cases or healthy controls, where 1.00 indicates perfect reliability (sensitivity/specificity) and 0.50 indicates no more information than a random guess. CKD cases were defined either as participants with eGFR <60 mL/min/1.73 m^2^ (CKD-G) or by the electronic health records (EHR) from the National Health Service (CKD-Diagnosed).

Model calibration was evaluated using graphical and quantitative approaches implemented in the *rms* package in R. Calibration plots were constructed by comparing predicted eGFR values with observed eGFR across the full range of predictions. A locally weighted smoothing (LOESS) curve was used to visualize agreement between predicted and observed values, with the 45-degree line representing perfect calibration. Calibration-in-the-large (CITL) was estimated by fitting a linear calibration model with observed eGFR as the outcome and predicted eGFR as the predictor, with the intercept reflecting systematic over- or under-prediction. A calibration slope was also estimated to assess whether predictions were overly extreme or insufficiently variable.

Decision curve analysis was performed (using the *rmda* package in R) to evaluate the potential clinical utility of each prediction model across a range of decision thresholds. Net benefit was calculated for threshold probabilities between 1% and 30% using standard methods. Net benefit is defined as the proportion of true-positive classifications minus the proportion of false-positive classifications weighted by the relative harm of a false positive versus a false negative, which is determined by the selected risk threshold. Formally, net benefit was calculated as

$$Net\;Benefit=\frac{TP}{N}-\frac{FP}{N}\times \frac{p_{t}}{1-p_{t}},$$

where *TP* and *FP* denote the number of true positives and false positives, respectively, *N* is the total sample size, and *p*_*t*_ is the threshold probability. Net benefit was compared across prediction models and against default strategies of treating all individuals or treating none. Confidence intervals for net benefit were estimated using bootstrap resampling.

## Results

### Genetic and clinical algorithm for CKD risk

We developed a genetic and clinical algorithm to identify those at risk of but undiagnosed with CKD. Three polygenic risk scores have been developed to predict eGFR (Khan et al., 2022; Sinnott-Armstrong et al., 2021; Yu et al., 2021) and one for uACR (Tanigawa et al., 2022). Each of these PRSs was evaluated to determine whether they should be included in the algorithm. In addition to genetics, CKD risk is affected by CRFs such as sex, age, body mass index (BMI), systolic blood pressure (SBP), smoking status, low-density lipoprotein (LDL), and glycated hemoglobin (HbA1C) (Akrawi et al., 2018; Tsai et al., 2016).

We first determined which of the three PRSs for eGFR had the best performance (Khan et al., 2022; Sinnott-Armstrong et al., 2021; Yu et al., 2021). Each was tested in a model including the PRS along with the seven CRFs. Of the three GFR PRSs that were tested, the PRS from Yu et al. (2021) performed best (Supplementary Table S1). The model had a correlation with eGFR of 63% and explained 39% of the variance. The algorithm had AUC values of 0.863, 0.631, 0.682, 0.809, and 0.797 for CKD-G, CKD-A, CKD-GA, CKD-G + A, and CKD-Diagnosed, respectively. The PRS from Yu et al. (2021) is hereafter referred to as “GFR PRS”.

In addition to eGFR, uACR (>30 mg/g) is used to diagnose CKD. We assessed the performance of the PRS for uACR in a model containing the PRS and seven CRFs. The model had a correlation with observed log(uACR) of 31% (95% CI: 28.6–33.7), explained 10% (95% CI: 8.2–11.4) of its variance, and had an AUC of 0.749 (95% CI: 0.744–0.755) for CKD-Diagnosed (Supplementary Table S1). However, the uACR PRS contributed little to the model as the seven CRFs alone also had an AUC of 0.749 (95% CI: 0.744–0.755) for CKD-Diagnosed.

**FIGURE 1:**
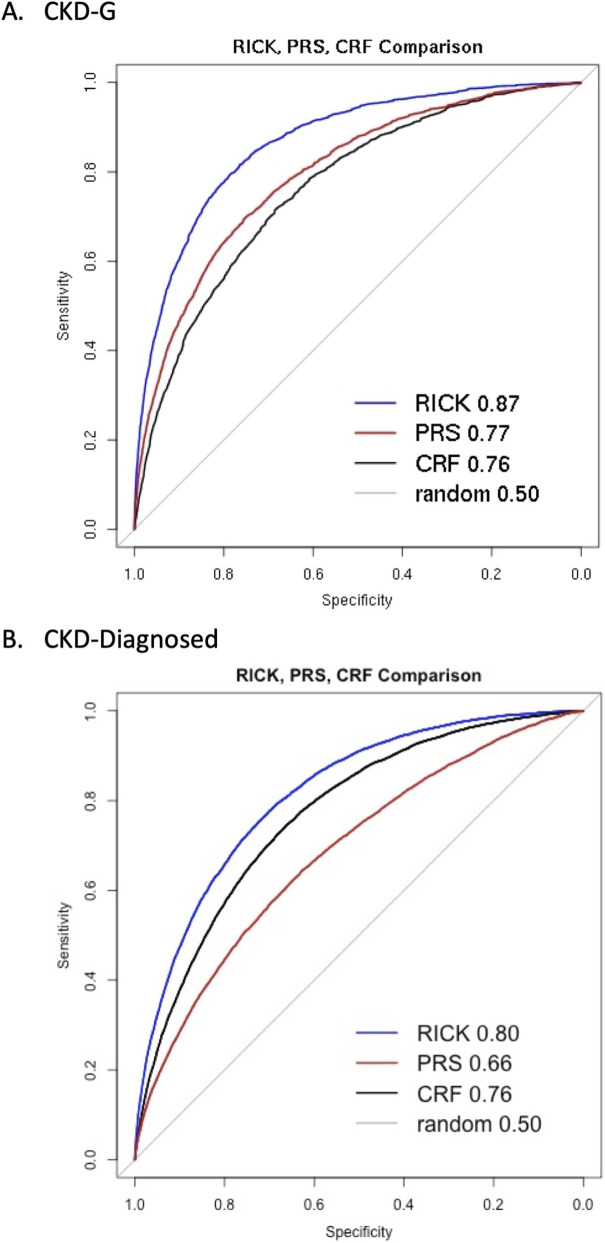
AUC plot for the prediction of CKD-G **(A)** and CKD-Diagnosed **(B)** by RICK, GFR PRS, and CRFs. The AUC for each predictor is shown.

We then generated a model containing the PRSs for both GFR and uACR as well as CRFs. This model had an AUC for predicting CKD-Diagnosed of 0.859 (95% CI: 0.853–0.864). Removing the PRS for uACR had a negligible effect on the performance of the model; when assessed on the 72,562 participants in the cohort that had both their GFR and uACR measured, the model had an AUC for CKD-Diagnosed of 0.856 (95% CI: 0.849–0.861). This indicates that the uACR PRS provided little contribution to predicting CKD-Diagnosed. In summary, the final model chosen included GFR PRSs and seven CRFs but not the uACR PRS (Figure 1). This model is hereafter referred to as “RICK” (RIsk for Chronic Kidney disease); its coefficients are listed in Supplementary Table S2.

**FIGURE 2:**
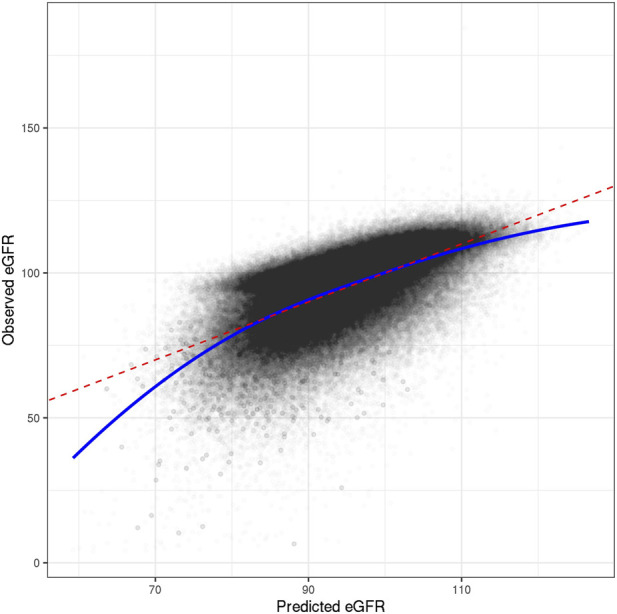
Comparison of the eGFR predicted by the RICK algorithm to the eGFR established from creatinine testing. Each point represents an individual participant, with darker regions indicating higher data density. The red dashed line represents the line of perfect calibration (predicted = observed). The blue curve shows the smoothed calibration curve (LOESS), illustrating the agreement between predicted and observed values across the range of predicted eGFR.

A comparison of the eGFR predicted by the RICK algorithm to the eGFR established from creatinine testing is shown in Figure 2. Calibration was excellent, with a calibration slope of 1.00 indicating appropriate prediction spread. Calibration-in-the-large was 0.10, suggesting only minimal systematic underprediction of eGFR. The calibration plot demonstrates reasonable agreement between predicted and observed eGFR in the central prediction range (80–105 ml/min/1.73 m^2^). However, the model tends to overestimate kidney function at both the lower and upper extremes, with observed eGFR falling below predicted values.

The performance of the RICK algorithm was further assessed with a Brier score, which measures the mean squared difference between predicted probability and observed outcome. The RICK model demonstrated the lowest Brier score (0.0161), indicating improved overall predictive accuracy compared with the CKD score (0.0167) and the clinical model (0.0173).

The RICK algorithm comprises a genetic test (GFR PRS) and seven clinical risk factors. We compared the performance of the two components (genetic test vs. all CRFs) by repeating the regression using each of the components by themselves (Table 1; Figure 1). The seven CRFs (sex, age, BMI, blood pressure, smoking status, LDL, and HbA1C) had a correlation of 44.9%, explained 20.2% of the variance in eGFR, and had AUC values of 0.764 and 0.760 for predicting CKD-G and CKD-Diagnosed status, respectively. Conversely, the GFR PRS by itself had a correlation of 43.0%, explained 18.5% of the variance in eGFR, and had AUC values of 0.765 and 0.660 for classifying CKD-G and CKD-Diagnosed status, respectively. These results indicate that the performance of GFR PRSs is similar to that of the CRFs at predicting eGFR and classifying CKD disease status. The best performance is obtained by combining both GFR PRSs and the CRFs in the RICK algorithm (Figure 1).

**TABLE 1:** Comparison of CRFs, PRSs, and RICK for predicting eGFR and CKD.

Predictive factor(s)	Performance			
​	Correlation (95% CI)	Variance explained (95% CI)	AUC CKD-G (95% CI)	AUC CKD-diagnosed
CRFs	0.449 (0.358–0.531)	0.202 (0.175–0.227)	0.764 (0.757–0.771)	0.760 (0.756–0.764)
GFR PRS	0.430 (0.386–0.480)	0.185 (0.145–0.224)	0.765 (0.789–0.802)	0.660 (0.656–0.665)
CRFs and GFR PRS	0.627 (0.585–0.672)	0.394 (0.337–0.456)	0.866 (0.860–0.871)	0.797 (0.794–0.800)

CRF, clinical risk factor; GFR PRS, chronic kidney disease polygenic risk score (Yu et al., 2021); Correlation, Spearman’s correlation; AUC, area under the curve.

CRFs: sex, age, BMI, SBP, smoking status, LDL, and HbA1C. All tests were performed with 244,671 participants with complete data for CRFs, and GFR PRSs.

**FIGURE 3:**
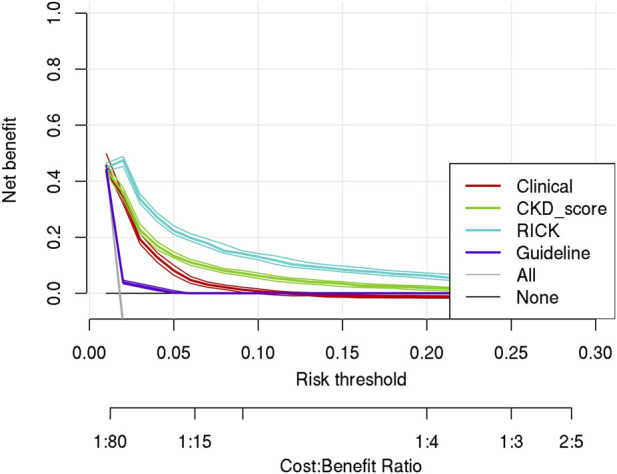
Decision curve analysis showing the net benefit of different prediction strategies across a range of risk thresholds. The x-axis represents the threshold probability used to define high risk, and the y-axis represents the net clinical benefit. Curves are shown for the RICK model (cyan), the clinical model using seven clinical risk factors (red), CKD score (green), and guideline-based strategy (purple). The thin lines represent 95% confidence intervals for the decision curves. The gray line represents the strategy of treating all individuals, and the black horizontal line represents treating none. Across most clinically relevant threshold probabilities, RICK provides the greatest net benefit compared with the clinical model, CKD score, and guideline-based strategy, indicating improved identification of individuals at elevated CKD risk while minimizing unnecessary interventions. The lower axis indicates the corresponding cost–benefit ratio associated with each threshold probability.

Decision curve analysis was used to evaluate the clinical utility of the prediction models (Figure 3). The RICK model demonstrated greater net benefit than the clinical model, CKD score, and guideline-based strategy across clinically relevant threshold probabilities. In particular, the RICK model showed the highest net benefit across thresholds of approximately 2%–20%, indicating improved identification of individuals at elevated CKD risk while minimizing unnecessary interventions.

**FIGURE 4:**
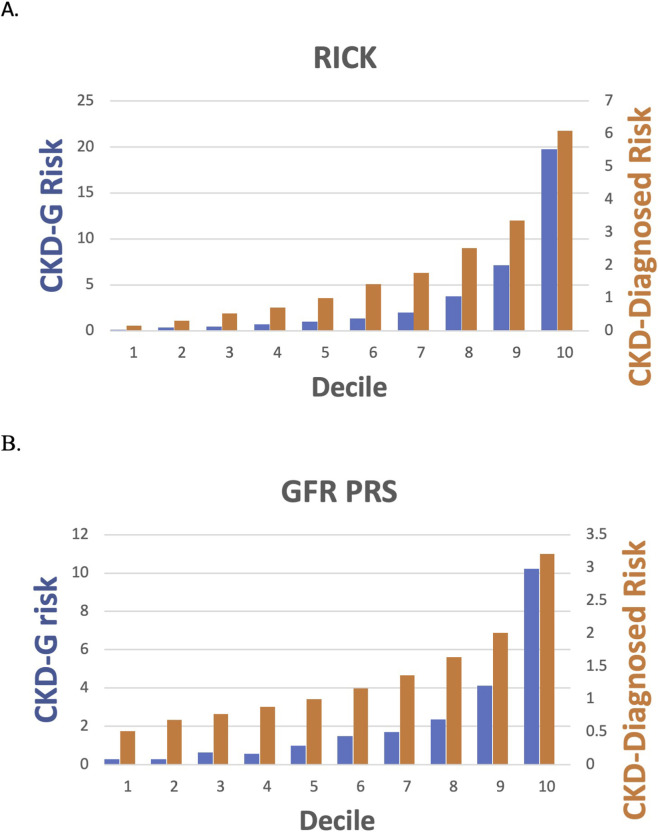
**(A)** CKD-G or CKD-Diagnosed risk (y-axis) for each decile of RICK (x-axis). In the highest RICK decile, the risk for CKD-G is 15.3-fold higher than the median with a prevalence of 18.1%, and the risk for CKD-Diagnosed is 4.8-fold higher with a prevalence of 16.2%. **(B)** CKD-G or CKD-Diagnosed risk for each decile of GFR PRS. In the highest GFR PRS decile, the risk for CKD-G is 8.8-fold higher than the median with a prevalence of 15.3%, and the risk for CKD-Diagnosed is 3.1-fold higher with a prevalence of 17.2%. Risk is normalized to the median.

The overall prevalence of CKD-G and CKD-Diagnosed in the cohort used to derive RICK was 1.7% and 7.7%. RICK stratifies the population into those with either higher or lower risk for CKD. Figure 4A shows the risk for CKD-G and CKD-Diagnosed for each decile of RICK. Participants in the highest decile of RICK had a prevalence for CKD-G (9.5%) that was 10.2-fold higher than those with median RICK scores. The prevalence for CKD-Diagnosed in the highest decile of RICK (26.8%) was 6.1-fold higher than the risk among those with median RICK scores. The highest decile of RICK from the United Kingdom Biobank contained 49.1% of all CKD-G cases (2,366 out of 4,812 total cases). Participants in the lowest decile of RICK had a prevalence of CKD-G of only 0.05%, 89% lower than those with median RICK scores. The lowest decile of RICK had a prevalence of CKD-Diagnosed of only 0.7%, which is 84% lower than those with median RICK scores.

Figure 4B shows how GFR PRS stratifies risk for CKD. Participants in the highest GFR PRS decile had a prevalence of 8.0% for CKD-G, 10.2-fold higher than those with median GFR PRSs. The prevalence for CKD-Diagnosed was 18.7%, 3.2-fold higher than those with median GFR PRSs. The highest decile of GFR PRSs from United Kingdom Biobank contained 2,170 cases of CKD-G, out of 4,812 total cases (45.0%). Participants in the lowest decile of GFR PRSs had a prevalence of CKD-G of 0.2%, 72% lower than those with median GFR PRSs. The lowest decile of GFR PRSs had a prevalence of CKD-Diagnosed of 2.9%, which is 49% lower than those with median GFR PRSs.

Table 2 shows the values of RICK and CKD PRS for each decile, along with the associated risk for CKD-G for each decile. This table enables an individual to use his or her RICK or CKD PRSs to appraise their risk.

**TABLE 2:** Values and CKD-G risk for each decile of RICK and CKD PRS.^a^

RICK decile	Range of RICK scores	Relative risk for CKD-G
1	<264.1	0.11
2	264.2 to 267.6	0.36
3	267.7 to 270.1	0.48
4	270.2 to 272.0	0.72
5	272.1 to 273.8	1
6	273.9 to 275.7	1.36
7	275.8 to 277.5	1.96
8	277.6 to 279.8	3.73
9	279.9 to 282.8	7.12
10	>282.9	19.7

CKD PRS decile	Range of CKD PRSs	Relative risk for CKD-G
1	<1.37	0.28
2	1.38 to 1.48	0.28
3	1.49 to 1.56	0.64
4	1.57 to 1.63	0.58
5	1.64 to 1.7	1
6	1.71 to 1.76	1.49
7	1.77 to 1.83	1.69
8	1.84 to 1.92	2.35
9	1.93 to 2.03	4.11
10	>2.04	10.23

^a^ Based on 262,044 participants from the United Kingdom Biobank.

If the general population were to have genotype data available, then RICK testing could identify those with high RICK scores and have them routinely tested for creatinine levels. To illustrate this, we show what would happen if RICK testing (but not universal creatinine testing) were used on the United Kingdom Biobank cohort. At the time of recruitment, 33,650 of 244,680 individuals had been previously diagnosed with CKD in their electronic health record via the National Health Service (Table 3). RICK testing would identify those in the highest decile, who have a 10.2-fold increased risk for low eGFR (Table 2). Although the United Kingdom Biobank performed creatinine testing on the entire cohort, this would not be the case for the general population. We show the results if creatinine testing were limited to just the highest decile of RICK; specifically, 4,398 of the 24,300 individuals (18%) in the highest RICK decile had low eGFR (<60 mL/min/1.73 m^2^) (Table 3). Of these, 1,895 were previously known to have CKD from there, and 2,503 would be newly diagnosed. Thus, if genotype data were available for the general population, RICK testing would increase the number of diagnosed cases of CKD by approximately 7.4%. RICK-positive individuals could undergo kidney function testing by either patient self-request or through recommendation from primary care.

**TABLE 3:** Identification of new CKD cases by RICK.

​	United Kingdom Biobank
Number of individuals	244,680
Number diagnosed with CKD in their EHRs via the NHS	33,650
Top decile RICK	24,468
Number with low eGFR from creatinine testing from the top RICK decile	4,398 (18%)
Number of cases of low eGFR that were not previously diagnosed in the EHRs	2,503 (57%)

### Genetic testing for patients known to have CKD

**FIGURE 5:**
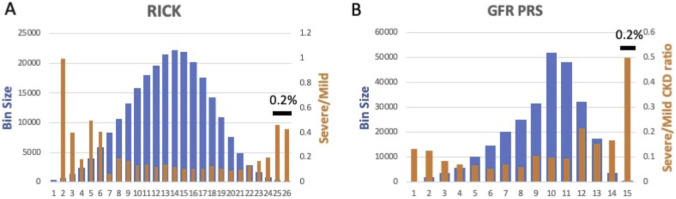
Ratios of severe/mild CKD cases (y-axis) for increasing values of RICK **(A)** or GFR PRS **(B)** shown on the x-axis in orange. Histograms of participants used to generate the algorithms are shown in blue. The fraction of the cohort showing increased severe/mild ratios is shown.

We examined whether RICK or the GFR PRS alone could provide information for treating patients already known to have CKD. In the United Kingdom Biobank cohort, 25,099 and 3,650 patients were diagnosed with mild (stage 3 but not severe) and severe (stages 4, 5, end stage renal disease, and renal transplant) CKD, respectively. If the algorithms were to provide information about the severity of CKD, one would expect that the relative number of patients with severe CKD would increase for high scores. We found that there was little change in the severe/mild ratio except at the extreme right-most tail of the distribution. For RICK and GFR PRS, patients with scores in the highest 0.2% had higher ratios of severe/mild CKD, respectively (Figure 5).

## Discussion

This study describes the development of a genetic and clinical algorithm (RICK) that may assist in identifying people with low eGFR, one of the main criteria for diagnosing CKD. RICK could play a role in improving renal healthcare because CKD is underdiagnosed in the general population. Early-stage CKD generally has no symptoms, and screening for CKD is typically performed only when indicated by age, hypertension, diabetes, or other risk factors. As a result, a large fraction of people with CKD are undiagnosed. Previous research estimated that 80%–90% of CKD cases are undetected (Shang et al., 2021; Verhave et al., 2014). The high fraction of undiagnosed cases of CKD poses both a current limitation and a future opportunity for improving public renal health.

One of the barriers to employing PRSs for CKD in the clinic is that they are interpreted in the context of a large population to determine relative risk for CKD—similar to assigning a grade on a curve. This necessitates access to the scores from a large cohort, which are not currently accessible for either individuals or for healthcare systems to use. In contrast, in this study, RICK can be used for an individual because the parameters for the RICK algorithm are provided, which allows CKD risk to be calculated.

The prevalence of CKD in the general population is too low to merit eGFR measurements for everyone. The RICK algorithm could be used to identify people at risk for low eGFR. They may be good candidates to have their creatinine levels tested to determine if they have low eGFR, which would enable prompt first-time access to renal healthcare. Based on the United Kingdom Biobank cohort, the highest RICK decile contained approximately 45% of all low eGFR (CKD-G) cases, and 8% of participants in this decile had low eGFR. A key point is that the RICK algorithm would mainly be used to identify people who should be screened with a serum creatinine test for CKD.

One barrier to using RICK is the cost of genotyping. As with the United Kingdom Biobank, the cost of genotyping could be spread over a large number of diseases. The benefit of RICK testing would be to identify new cases of CKD, thereby enabling access to renal healthcare. With United Kingdom Biobank, 2,503 new cases of CKD could potentially be diagnosed by RICK testing from a cohort of approximately 240,000. This extrapolates to approximately 2.6 million new cases in the US and 550,000 new cases in the United Kingdom.

Those identified as at risk for CKD could be most appropriately screened with urine protein quantification and measurement of serum creatinine and/or cystatin C on a recurring basis through their primary care physician and referred to nephrology when there is evidence for disease. Treatment options include well-accepted interventions that have been associated with reduced risk of CKD such as managing hypertension, glycemia, dyslipidemia, and weight loss (Luyckx et al., 2020; Jafar et al., 2016). Specifically, angiotensin-converting enzyme inhibitor/angiotensin-receptor blockers, (Jafar et al., 2001; Maione et al., 2011) inhibitors of the sodium glucose cotransporter-2, (The EMPA-KIDNEY Collaborative Group, 2023; Chertow et al., 2021; The Nuffield Department of Population Health Renal Studies Group and the SGLT2 inhibitor Meta-Analysis Cardio-Renal Trialists’ Consortium, 2022), and finerenone (Theodorakopoulou and Sarafidis, 2024) have been shown to slow the progression of kidney disease (Luyckx et al., 2020). Overall, preventative treatment from nephrology specialists has been shown to reduce progression of CKD compared to treatment from general practitioners (Black et al., 2010).

In the RICK algorithm, the GFR PRS provides an equivalent amount of information compared to the seven clinical risk factors for risk for CKD. Specifically, the GFR PRS explained 18.5% of the variance in eGFR, while a combination of all of the CRFs explained 20.2%. CRFs such as age, hypertension (blood pressure), and hyperglycemia (HbA1C) are well-accepted measures of CKD risk (Tsai et al., 2016; Obrador et al., 2011). GFR PRS could also be used as a CKD risk factor since it is as informative than those in current use.

The two main criteria for diagnosing CKD are low eGFR and high uACR. RICK is informative for low eGFR but not for high uACR. Little or no information was added by including either observed uACR or a PRS for uACR in models for predicting CKD (“Methods”). Thus, RICK is most useful for identifying cases of CKD due to low GFR (CKD-G) rather than high uACR.

Because it is a genetic test, GFR PRS is constant throughout life, unlike clinical risk factors that are most informative for older patients. In principle, the GFR PRS could be used to identify people with lifelong risk for CKD when they are young or middle-aged. By itself, GFR PRS has an AUC for predicting CKD-G of 0.866, which is a measurement of how well the PRS can distinguish CKD cases from controls. Although young people with a high GFR PRS would likely to not yet have CKD, they would be more likely to develop CKD with age. Identification of those at risk may facilitate monitoring and early intervention.

Steinbrenner et al. (2023) also assessed whether a related PRS for GFR was informative in treating patients with mild CKD. In their study, patients with mild CKD were followed over a 6-year timeframe, during which 9.5% of patients developed kidney failure. The top decile of that PRS had a hazard ratio of 1.5 for predicting kidney failure. They also found that the PRS did not improve performance over the four-factor kidney failure risk equation, an algorithm used in research studies to predict kidney failure based on age, sex, eGFR and uACR (Tangri et al., 2011). Our data also suggested that those with severe versus mild CKD did not differ in RICK and GFR PRS, except for a tiny proportion of people at the extreme tail of the distribution. Thus, RICK and the GFR PRS may be more effective at identifying undiagnosed patients that would benefit from testing rather than stratifying patients with established CKD.

One limitation of this study is that the cohort only included participants of European ancestry. The GFR PRS by Yu et al. (2021) used in this study is highly similar (97% correlation) to the PRS used by Khan et al. (2022), who showed that the top 2% of their GFR PRS conveyed similar increases in CKD risk across all ancestry groups, suggesting that these results may be more widely applicable.

Second, RICK is not predictive for uACR, which is another criterion for diagnosing CKD. Therefore, the RICK algorithm adds little value for detecting CKD defined by elevated uACR (albuminuria), which is a major criterion for diagnosing CKD (e.g., in diabetic kidney disease). Future research may improve the PRS for uACR such that it could be incorporated into RICK.

Third, approximately 39% of the cohort used to derive GFR PRS was from the United Kingdom Biobank (Yu et al., 2021). The GFR PRS and RICK algorithms were validated on 10% of the United Kingdom Biobank population that was held out from the development step. Nevertheless, it is possible that their performances are inflated as there is overlap between the cohorts used to derive GFR PRS in the first place and validation of the algorithms in this study (Yu et al., 2021).

Fourth, the participants in United Kingdom Biobank may not accurately reflect the general population, which would affect estimates of CKD prevalence. For instance, United Kingdom Biobank participants were older than the general population.

A fifth limitation is that information on electronic medical records may be incomplete. Therefore, when no CKD code is found in the EHRs, this could mean either that CKD is undiagnosed or is at least not documented as such.

Sixth, a solid diagnosis of CKD should be defined using more than a single measurement of eGFR and/or uACR; single measurements may show that fluctuation and classification of CKD-G based on them may lead to misclassification and both false positives and false negatives. Specifically, a single eGFR measurement that is lower than the actual eGFR would be misclassified as normal, and a single eGFR measure that is higher would be misclassified as having CKD rather than being normal.

A further limitation is that the diagnosis of CKD in electronic health records and the measurement of clinical risk factors may not have happened at the same time. We did not have information about medications, which may affect some measurements of clinical risk factors and diagnosis outcomes that are relevant for the analyses we performed. The date when the ICD codes were entered into the electronic health records is not known, while the clinical risk factors were measured together during the initial assessment by the United Kingdom Biobank from 2006 to 2010.

Finally, clinical risk factors such as blood pressure and HbA1C are influenced by medications (e.g., antihypertensives and glucose-lowering drugs), which were not taken into account by the RICK algorithm. The presence and duration of these medications taken by the individuals in the training set could skew how the HbA1C and SBP coefficients were derived during the training phase of RICK.

A major issue in using an algorithm that includes a PRS is the cost of genetic testing. Despite dramatic decreases in cost, the cost for universal population genetic screening would still be high and potentially unfavorable compared with testing for laboratory markers of CKD. This, however, will not be a barrier if the genetic information has already been obtained for other, diverse purposes, as is increasingly the case. Many millions of people have already had their genotypes determined, either through direct-to-consumer DNA testing companies or through their healthcare providers. As genotypes become more available, it will be possible to calculate PRSs for many types of chronic disease, including CKD, and thus spread the cost of genetic testing across many genetic diseases. It may thus be reasonable to use existing genetic data for the general population to identify those at risk for CKD and thereby enable selective screening and early intervention. Genetic screening would facilitate the early identification of individuals at risk of CKD throughout their lifetime, long before other relevant clinical risk factors may be present. Careful decision- and cost-effectiveness analysis is needed to assess the relative utility of different screening strategies in different settings and with different cost assumptions.

A second major issue for the utility of such genetic screening is whether it can lead to better outcomes. This will need to be demonstrated in prospective studies. Nevertheless, given the potentially substantial benefit of early intervention in terms of slowing disease progression along with the growing armament of therapeutics available to impact both CKD and associated cardiovascular disease, identification of populations at risk may have a favorable impact both for individual patients as well as for policymakers managing at-risk populations.

In conclusion, this study describes the RICK genetic algorithm that could be used to aid in identifying new cases of chronic kidney disease based on low eGFR. This could facilitate early access to renal healthcare and either delay or prevent the progression of the disease.

## Data Availability

Data used in the published polygenic risk scores described in this paper are available at https://www.pgscatalog.org/ using the PGS codes shown in Supplementary Table S1. Coefficients for the RICK algorithm are shown in Supplementary Table S2.
