# Supplementary material for: A genetic and clinical risk factor algorithm to aid in identifying new cases of chronic kidney disease from the general population
Source: Front Genet. 2026 Jul 9;17:1799312. doi: 10.3389/fgene.2026.1799312 (PMC13391044; doi:10.3389/fgene.2026.1799312)
Supplement: Supplementary file 1 [file Table1.docx]

Supplemental Table 1. Comparison of different PRS's for predicting CKD risk.

|  | eGFR | | AUC | | | | |  |
| --- | --- | --- | --- | --- | --- | --- | --- | --- |
| PRS + CRFs | R | R2 | CKD-G | CKD-A | CKD-GA | CKD-G+A | CKD-Diagnosed | PRS Ref. |
| PGS000884 | 0.63 | 0.39 | 0.863  (0.860-0.871) | 0.631  (0.625 to 0.638) | 0.682  (0.677-0.687) | 0.809  (0.800-0.818) | 0.797  (0.794-0.800) | 20 |
| PGS002237 | 0.61 | 0.37 | 0.851  (0.845-0.857) | 0.631  (0.625 to 0.637) | 0.652  (0.647 to 0.658) | 0.797  (0.784 to 0.810) | 0.793  (0.790-0.796) | 17 |
| PGS000682 | 0.58 | 0.34 | 0.828  (0.822-0.834) | 0.632  (0.626 to 0.638) | 0.650  (0.645 to 0.655) | 0.772  (0.758 to 0.786) | 0.786  (0.783-0.790) | 18 |
|  |  |  |  |  |  |  |  |  |
|  | uACR | |  |  |  |  |  |  |
|  | R | R2 |  |  |  |  |  |  |
| PGS001107 | 0.31 | 0.10 | ND | 0.660  (0.655 to 0.666) | 0.652  (0.647 to 0.658) | 0.721  (0.706 to 0.735) | 0.749  (0.744-0.755) | 19 |

eGFR, estimated glomerular filtration rate; AUC, area under the curve, uACR, urinary albumin to creatine ratio. PRS codes are from the PGS catalog (https://www.pgscatalog.org/). R and R2 are the correlation and variance explained for eGFR. CKD-G (eGFR < 60 mL/min/1.73 m2), CKD-A (uACR > 30 mg/g), CKD-GA (CKD-G or CKD-A), CKD-G+A (CKD-G and CKD-A), CKD-EHR (CKD coded in electronic health records)
