# Supplementary material for: A genetic and clinical risk factor algorithm to aid in identifying new cases of chronic kidney disease from the general population
Source: Front Genet. 2026 Jul 9;17:1799312. doi: 10.3389/fgene.2026.1799312 (PMC13391044; doi:10.3389/fgene.2026.1799312)
Supplement: Supplementary file 2 [file Table2.docx]

Supplemental Table 2. Coefficients for the RICK algorithm.

|  | Estimate | SD |
| --- | --- | --- |
| (Intercept) | 173.83 | 0.2658 |
| Sex | 0.4196 | 0.04313 |
| Age | -0.7558 | 0.00282 |
| BMI | -0.2096 | 0.004755 |
| SBP | 0.0240 | 0.001071 |
| smoke | 2.1876 | 0.07965 |
| LDL | 0.4677 | 0.02459 |
| HbA1C | 0.02468 | 0.003503 |
| GFR PRS | -21.67 | 0.08108 |

Sex (F=0, M=1); BMI, body mass index (kg/m2); SBP, systolic blood pressure (mmHg); HbA1C (mmol/mol); LDL, low density lipoprotein (mmol/L); GFR PRS, GFR polygenic risk score.^20^ All coefficients were significant at p < 1E-16.
