# Supplementary material for: A genetic and clinical risk factor algorithm to aid in identifying new cases of chronic kidney disease from the general population
Source: Front Genet. 2026 Jul 9;17:1799312. doi: 10.3389/fgene.2026.1799312 (PMC13391044; doi:10.3389/fgene.2026.1799312)
Supplement: Supplementary file 4 [file Table3.docx]

Supplemental Table 3. EHR codes used to identify cases of CKD-Diagnosed from the NHS.

| ICD-10 | Description | Participants | CKD severity |
| --- | --- | --- | --- |
| N18.0 | End-stage renal disease | 461 | Severe |
| N18.1 | Chronic kidney disease, stage 1 | 245 | Mild |
| N18.2 | Chronic kidney disease, stage 2 | 1964 | Mild |
| N18.3 | Chronic kidney disease, stage 3 | 15,125 | Mild |
| N18.4 | Chronic kidney disease, stage 4 | 2,024 | Severe |
| N18.5 | Chronic kidney disease, stage 5 | 1,562 | Severe |
| Y84.1 | Kidney dialysis | 307 | Severe |
| M01 | Transplantation of kidney | 17 | Severe |
| N18.9 | Chronic kidney disease, unspecified | 12012 |  |
|  |  |  |  |
| Read2 |  |  |  |
| 1Z10 | Chronic kidney disease stage 1 | 201 | Mild |
| 1Z11 | Chronic kidney disease stage 2 | 977 | Mild |
| 1Z12 | Chronic kidney disease stage 3 | 2,626 | Mild |
| 1Z13 | Chronic kidney disease stage 4 | 153 | Severe |
| 1Z14 | Chronic kidney disease stage 5 | 44 | Severe |
| 1Z15 | Chronic kidney disease stage 3A | 232 | Mild |
| 1Z16 | Chronic kidney disease stage 3B | 54 | Mild |
| 1Z17 | Chronic kidney disease stage 1 with proteinuria | 1 | Mild |
| 1Z18 | Chronic kidney disease stage 1 without proteinuria | 5 | Mild |
| 1Z19 | Chronic kidney disease stage 2 with proteinuria | 9 | Mild |
| 1Z1a | CKD G4A1 - chronic kidney disease with glomerular filtration rate category G4 and albuminuria category A1 | 64 | Severe |
| 1Z1b | CKD G4A2 - chronic kidney disease with glomerular filtration rate category G4 and albuminuria category A2 | 23 | Severe |
| 1Z1c | CKD G4A3 - chronic kidney disease with glomerular filtration rate category G4 and albuminuria category A3 | 2 | Severe |
| 1Z1d | CKD G5A1 - chronic kidney disease with glomerular filtration rate category G5 and albuminuria category A1 | 22 | Severe |
| 1Z1e | CKD G5A2 - chronic kidney disease with glomerular filtration rate category G5 and albuminuria category A2 | 243 | Severe |
| 1Z1f | CKD G5A3 - chronic kidney disease with glomerular filtration rate category G5 and albuminuria category A3 | 8 | Severe |
| 1Z1A | Chronic kidney disease stage 2 without proteinuria | 64 | Mild |
| 1Z1B | CKD stage 3 with proteinuria | 23 | Mild |
| 1Z1C | CKD stage 3 without proteinuria | 101 | Mild |
| 1Z1D | CKD stage 3A with proteinuria | 22 | Mild |
| 1Z1E | CKD stage 3A without proteinuria | 243 | Mild |
| 1Z1F | Chronic kidney disease stage 3B with proteinuria | 8 | Mild |
| 1Z1G | CKD stage 3B without proteinuria | 46 | Mild |
| 1Z1H | Chronic kidney disease stage 4 with proteinuria | 8 | Severe |
| 1Z1J | Chronic kidney disease stage 4 without proteinuria | 11 | Severe |
| 1Z1K | Chronic kidney disease stage 5 with proteinuria | 3 | Severe |
| 1Z1L | Chronic kidney disease stage 5 without proteinuria | 1 | Severe |
| 1Z1M | CKD G1A1 - chronic kidney disease with glomerular filtration rate category G1 and albuminuria category A1 | 1 | Mild |
| 1Z1X | CKD G3bA1 - chronic kidney disease with glomerular filtration rate category G3b and albuminuria category A1 | 3 | Mild |
| 1Z1Z | CKD G3bA3 - chronic kidney disease with glomerular filtration rate category G3b and albuminuria category A3 | 2 | Mild |
| 1Z1T | CKD G3aA1 - chronic kidney disease with glomerular filtration rate category G3a and albuminuria category A1 | 11 | Mild |
| K051 | Chronic kidney disease stage 1 | 4 | Mild |
| K052 | Chronic kidney disease stage 2 | 8 | Mild |
| K053 | Chronic kidney disease stage 3 | 242 | Mild |
| K054 | Chronic kidney disease stage 4 | 9 | Severe |
| K055 | Chronic kidney disease stage 5 | 2 | Severe |
| K050 | End stage renal failure | 14 | Severe |
| 14V2 | H/O: kidney dialysis | 5 | Severe |
| 7L1A | Dialysis for renal failure | 46 | Severe |
| 4678 | Proteinuria | 235 |  |
|  |  |  |  |
| Read3 |  |  |  |
| X30In | Chronic kidney disease | 371 |  |
| XaLHG | Chronic kidney disease stage 1 | 263 | Mild |
| XaLHH | Chronic kidney disease stage 2 | 1880 | Mild |
| XaLHI | Chronic kidney disease stage 3 | 6,034 | Mild |
| XaLHJ | Chronic kidney disease stage 4 | 232 | Severe |
| XaLHK | Chronic kidney disease stage 5 | 75 | Severe |
| XaNbn | Chronic kidney disease stage 3A | 680 | Mild |
| XaNbo | Chronic kidney disease stage 3B | 106 | Mild |
| XaO3p | Chronic kidney disease stage 1 with proteinuria | 21 | Mild |
| XaO3q | Chronic kidney disease stage 1 without proteinuria | 36 | Mild |
| XaO3r | Chronic kidney disease stage 2 with proteinuria | 72 | Mild |
| XaO3s | Chronic kidney disease stage 2 without proteinuria | 504 | Mild |
| XaO3t | Chronic kidney disease stage 3 with proteinuria | 211 | Mild |
| XaO3u | Chronic kidney disease stage 3 without proteinuria | 1648 | Mild |
| XaO3v | Chronic kidney disease stage 3 with proteinuria | 159 | Mild |
| XaO3w | Chronic kidney disease stage 3A without proteinuria | 1256 | Mild |
| XaO3x | Chronic kidney disease stage 3B with proteinuria | 67 | Mild |
| XaO3y | Chronic kidney disease stage 3B without proteinuria | 206 | Mild |
| XaO3z | Chronic kidney disease stage 4 with proteinuria | 72 | Severe |
| XaO41 | Chronic kidney disease stage 5 with proteinuria | 25 | Severe |
| XaO42 | Chronic kidney disease stage 5 without proteinuria | 10 | Severe |
| XaXTz | History of chronic kidney disease | 7 |  |
| Xac9y | CKD G1A1 - chronic kidney disease with glomerular filtration rate category G1 and albuminuria category A1 | 1 | Mild |
| XacA2 | CKD G1A3 - chronic kidney disease with glomerular filtration rate category G1 and albuminuria category A3 | 2 | Mild |
| XacA4 | CKD G2A1 - chronic kidney disease with glomerular filtration rate category G2 and albuminuria category A1 | 6 | Mild |
| XacA9 | CKD G2A3 - chronic kidney disease with glomerular filtration rate category G2 and albuminuria category A3 | 1 | Mild |
| XacAm | CKD G3aA1 - chronic kidney disease with glomerular filtration rate category G3a and albuminuria category A1 | 51 | Mild |
| XacAN | CKD G3aA2 - chronic kidney disease with glomerular filtration rate category G3a and albuminuria category A2 | 3 | Mild |
| XacAO | CKD G3aA3 - chronic kidney disease with glomerular filtration rate category G3a and albuminuria category A3 | 7 | Mild |
| XacAV | CKD G3bA1 - chronic kidney disease with glomerular filtration rate category G3b and albuminuria category A1 | 5 | Mild |
| XacAW | CKD G3bA1 - chronic kidney disease with glomerular filtration rate category G3b and albuminuria category A1 | 3 | Mild |
| XacAX | CKD G3bA1 - chronic kidney disease with glomerular filtration rate category G3b and albuminuria category A1 | 5 | Mild |
| XacAb | CKD G4A1 - chronic kidney disease with glomerular filtration rate category G4 and albuminuria category A1 | 1 | Severe |
| XacAd | CKD G4A2 - chronic kidney disease with glomerular filtration rate category G4 and albuminuria category A2 | 3 | Severe |
| XacAe | CKD G4A3 - chronic kidney disease with glomerular filtration rate category G4 and albuminuria category A3 | 1 | Severe |
|  |  |  |  |
|  | CKD-Diagnosed total | 33,650 |  |

Participants indicates the number of unique individuals with the diagnostic code from UK Biobank.
